# Effect of Device Scaling on Electron Mobility in Nanoscale GaN HEMTs with Polarization Charge Modulation

**DOI:** 10.3390/nano12101718

**Published:** 2022-05-18

**Authors:** Peng Cui, Yuping Zeng

**Affiliations:** 1 Institute of Novel Semiconductors, Shandong University, Jinan 250100, China; 2 Department of Electrical and Computer Engineering, University of Delaware, Newark, DE 19716, USA

**Keywords:** GaN HEMTs, scaling, electron mobility, scattering, polarization charge

## Abstract

We have experimentally investigated the impact of vertical and lateral scaling on low-field electron mobility (*µ*) in InAlN/GaN high-electron-mobility transistors (HEMTs). It is found that *µ* reduces as InAlN barrier (*T*_B_) and gate length (*L*_G_) scale down but increases with the scaled source–drain distance (*L*_SD_). Polarization Coulomb Field (PCF) scattering is believed to account for the scaling-dependent electron mobility characteristic. The polarization charge distribution is modulated with the vertical and lateral scaling, resulting in the changes in *µ* limited by PCF scattering. The mobility characteristic shows that PCF scattering should be considered when devices scale down, which is significant for the device design and performance improvement for RF applications.

## 1. Introduction

Due to the high breakdown voltage, high two-dimensional electron gas densities, and high electron saturation velocity, gallium nitride (GaN) high-electron-mobility transistors (HEMTs) have been ideal for high-frequency and high-power applications, such as radar communications, electronic countermeasures, 5G applications, small base stations, new communication microsatellites, power transmission and automotive electronics [1,2,3,4,5]. Yan Tang et al. fabricated the AlN/GaN/AlGaN double heterojunction HEMTs with fully passivation and n^+^-GaN ohmic contact regrowth technology, demonstrating a record high current/power gain cutoff frequency *f*_T_/*f*_max_ of 454/444 GHz on a 20 nm-gate-length HEMT with gate–source and gate–drain spacings of 50 nm [6]. Jeong-Gil Kim et al. reported an AlGaN/GaN HEMT structure on the high-quality undoped thick AlN buffer layer with a high breakdown voltage of 2154 V and a very high figure of merit (FOM) of ~1.8 GV^2^·Ω^−1^·cm^−2^ [7]. Xiaoyu Xia et al. reported a new type of AlGaN/GaN HEMTs with a microfield plate (FP) with a breakdown voltage increase from 870 V to 1278 V by adjusting the distribution of the potential and channel electric field [8]. Maddaka Reddeppa et al. demonstrated high photoresponse and the electrical transport properties of a pristine GaN nanorod-based Schottky diode with an optimized Schottky barrier height [9]. Kedhareswara Sairam Pasupuleti et al. developed the integration of conductive polypyrrole (Ppy) and GaN nanorods for high-performance self-powered UV-A photodetectors, exhibiting superior photoresponse properties such as detectivity, responsivity, external quantum efficiency, good stability and reproducibility [10].

To further improve device performance, device scaling in GaN HEMTs is necessary [6,11,12]. The effects of scaling on short-channel effects (SECs), leakage current, electron velocity, frequency characteristics have been studied [13,14,15,16,17,18], providing insightful guidance for device design and performance improvement. However, few studies about the impact of scaling on electron mobility have been reported. In general, low-field mobility should not change when devices scale down. However, due to the spontaneous and piezoelectric polarization in GaN HEMTs, there are polarization charges in the barrier layer [19,20], which is different from conventional transistors (Si, GaAs, et al.). The change in the polarization charge distribution is related to the device dimension and can result in scattering on the channel electrons [21,22], which leads to a possible change in mobility with device scaling. In this article, to demonstrate this influence, the InAlN/GaN HEMTs with various barrier thicknesses, source–drain distances, and gate lengths are fabricated and the effect of scaling on electron mobility is studied.

## 2. Experiment

**Figure 1:**
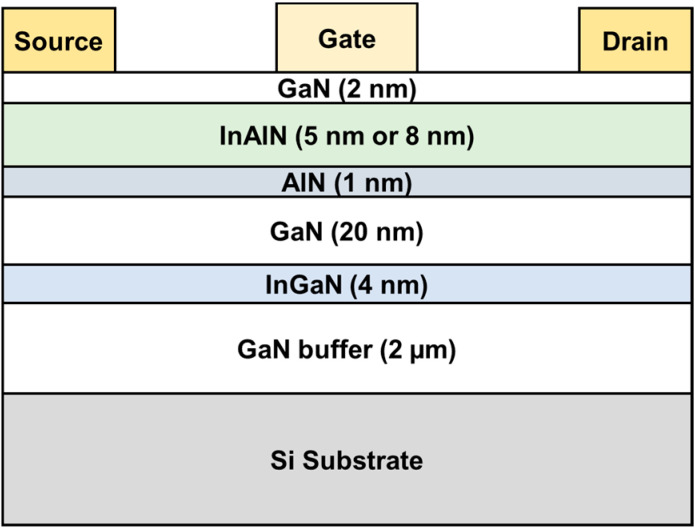
Schematic cross-section of the fabricated InAlN/GaN HEMT with two different InAlN barrier thickness (8 nm and 5 nm, respectively).

The lattice-matched In_0.17_Al_0.83_N/GaN HEMT structure is grown by metal–organic chemical vapor deposition on a Si substrate, as shown in Figure 1, consisting of a 2 nm GaN cap, an InAlN barrier, a 1 nm AlN interlayer, a 15 nm GaN channel layer, a 4 nm In_0.12_Ga_0.88_N back-barrier and a 2 μm undoped GaN buffer. Here, two different InAlN layers with the thicknesses of 8 nm and 5 nm are grown. The device process started with mesa isolation with Cl_2_-based inductively coupled plasma (ICP) etching. Then, Ohmic contact was formed with Ti/Al/Ni/Au metal deposition and annealed at 850 °C for 40 s. Ni/Au gate Schottky contact was deposited in the center of the source–drain region to complete the process. For the large devices, the gate length (*L*_G_), gate–source distance (*L*_GS_), and gate–drain distance (*L*_GD_) of the devices are all 2 µm. For the RF devices, two types of devices are fabricated. For type I, *L*_G_ of the devices is fixed at 50 nm and *L*_SD_ is 2, 1, and 0.6 µm, respectively. For type II, *L*_SD_ of the devices is fixed at 1 µm and *L*_G_ is 50, 100, and 150 nm, respectively. Here, the gate of all the devices is located between the source and drain regions, and the gate width is 2 × 20 µm. The current–voltage (*I*–*V*) and capacitance–voltage (*C*–*V*) measurements were carried out by using an Agilent B1500A semiconductor parameter analyzer (Agilent Technologies, Santa Clara, CA, USA).

## 3. Results and Discussion

**Figure 2:**
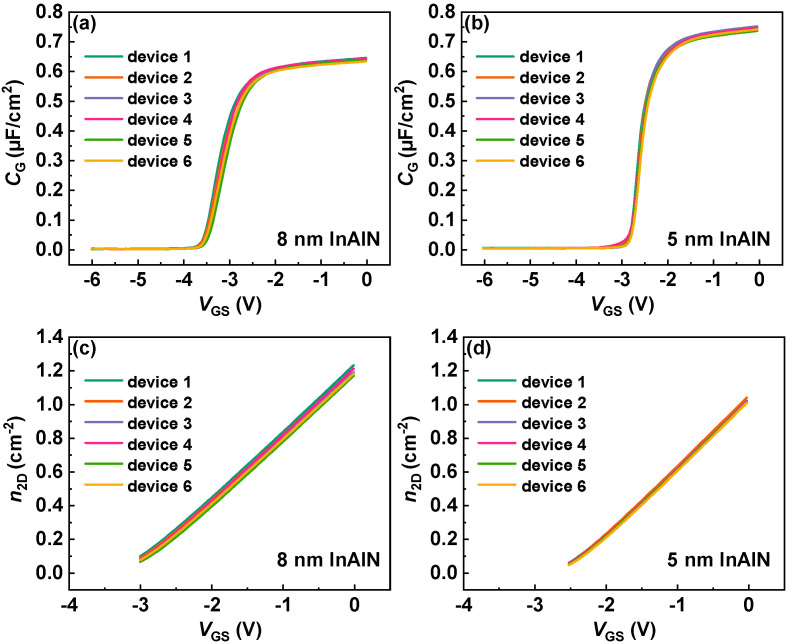
Gate capacitance (*C*_G_) of the InAlN/GaN diode with (**a**) 8 nm InAlN and (**b**) 5 nm InAlN, respectively. Two-dimensional electron gas electron density (*n*_2D_) of the InAlN/GaN diode with (**c**) 8 nm InAlN and (**d**) 5 nm InAlN, respectively.

**Figure 3:**
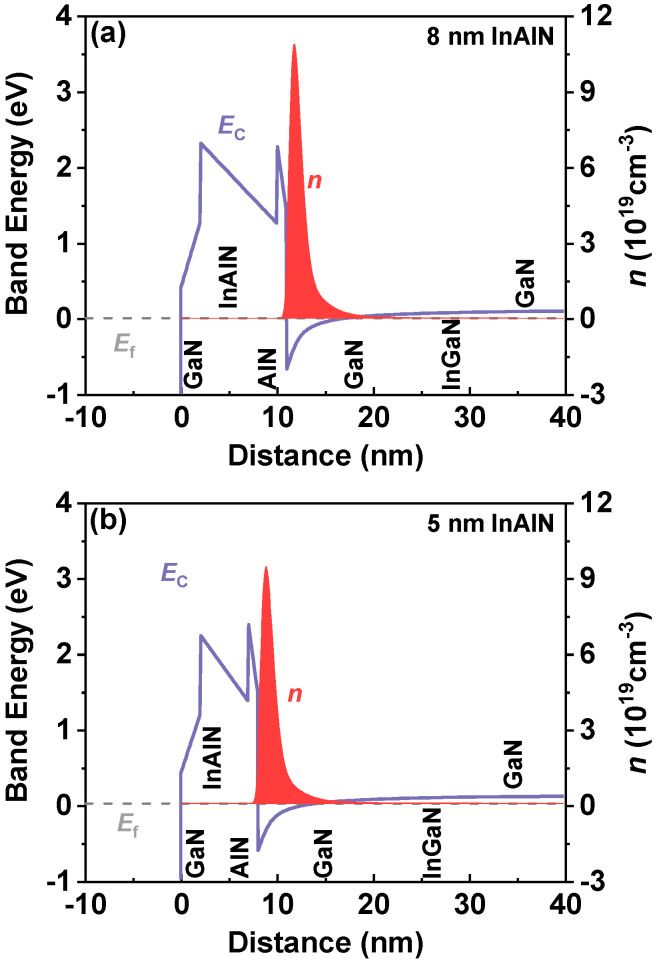
Simulated band structure and 2DEG electron density as a function of the distance from the material surface of the InAlN/GaN heterostructure with (**a**) 8 nm InAlN and (**b**) 5 nm InAlN, respectively.

Figure 2a,b show the measured capacitances (*C*) of the InAlN/GaN circle diodes with both InAlN barrier thicknesses (*T*_B_). Here, six devices are measured and a good consistency is presented. An improved *C* and a subthreshold voltage (*V*_T_) shift are observed due to the reduced InAlN barrier thickness (*C* = *ε*/*T*_B_, *ε* is the dielectric constant of InAlN barrier). Through integrating C-V curves, electron density (*n*_2D_) is extracted as shown in Figure 2c,d. It shows that the InAlN/GaN heterostructure with 8 nm InAlN barrier presents higher electron density. Figure 3 shows the simulated band structure and 2DEG electron density as a function of the distance from the material surface of the InAlN/GaN heterostructure, which is calculated by self-consistently solving Schrodinger’s and Poisson’s equations [23,24]. Compared with the 5 nm InAlN barrier, the InAlN/GaN heterostructure with an 8 nm InAlN barrier also shows a higher electron density peak. In GaN HEMTs, the surface states are identified as the source of channel electrons. Due to the spontaneous polarization filed, the increase in InAlN barrier thickness can increase the energy of the surface states, resulting in higher electron density [25,26].

**Figure 4:**
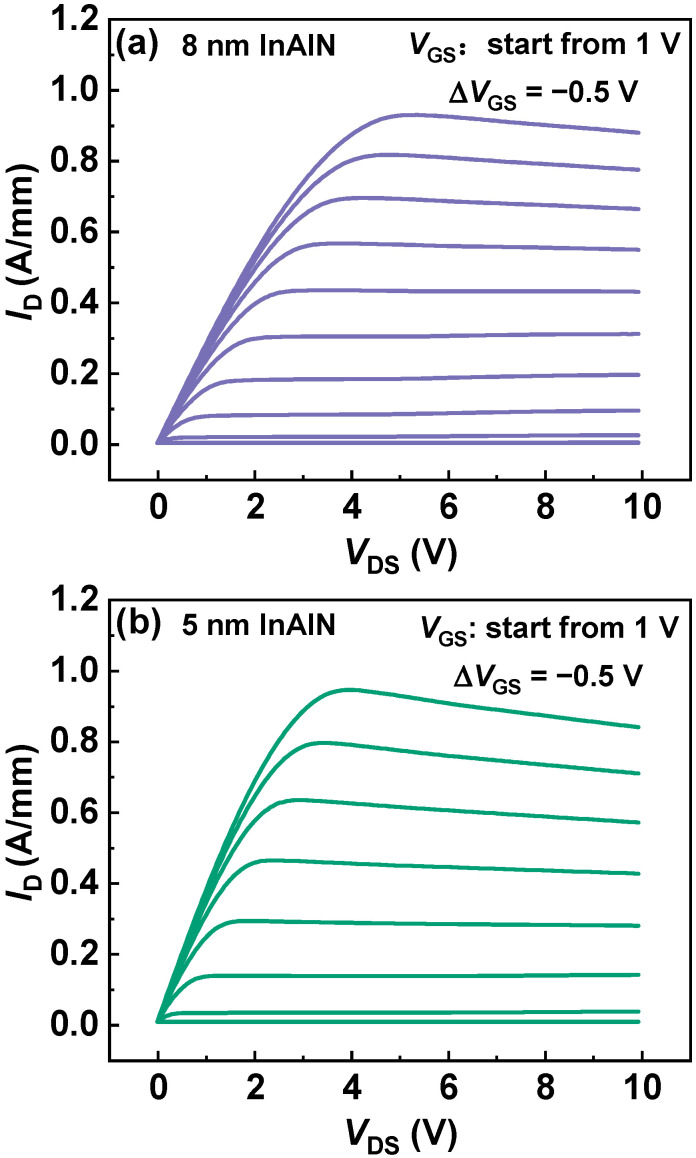
Output characteristics of the InAlN/GaN HEMTs with (**a**) 8 nm InAlN and (**b**) 5 nm InAlN, respectively.

Figure 4 shows the output characteristics of the InAlN/GaN HEMTs with different InAlN thickness. The *L*_G_, *L*_GS_, and *L*_GD_ of the devices are all 2 µm. To extract low-field mobility, the drain current (*I*_D_) at *V*_DS_ = 0.1 V in the output characteristics are used. At *V*_GS_ = 0 V, the total source–drain resistance (*R*_SD_) can be written as

(1) $$R_{SD}=\frac{V_{DS}}{I_{DS}}=2R_{C}+\frac{L_{G}+L_{GS}+L_{GD}}{n_{2D0}q\mu_{0}}$$

where *R*_C_ is the ohmic contact resistance, *q* is the electron charge, and *µ*_0_ and *n*_2D0_ are the electron mobility and electron density under the gate region with *V*_GS_ = 0 V. Here, only *µ*_0_ and *R*_C_ are unknown. Electron mobility in GaN HEMTs is limited by polar optical phonon (*µ*_POP_), polarization Coulomb field (*µ*_PCF_), acoustic phonon (*µ*_AP_), interface roughness (*µ*_IFR_), and dislocation (*µ*_DIS_) scatterings [22,27,28]. PCF scattering is related to the nonuniformity of polarization charge distribution [21,22]. At *V*_GS_ = 0 V, the polarization charge distribution is uniform, and the PCF can be neglected. Based on the two-dimensional (2D) scattering theory and the obtained *n*_2D0_ [27], *µ*_0_ can be calculated with 1/*µ*_0_ = 1/*µ*_POP_ + 1/*µ*_AP_ + 1/*µ*_IFR_ + 1/*µ*_DIS_, and then *R*_C_ can be determined with (1). Based on the obtained *n*_2D0_ and *µ*_0_, the electron mobility *µ* under the gate region under different *V*_GS_ can be extracted from

(2) $$\frac{V_{DS}}{I_{DS}}=2R_{C}+\frac{L_{G}}{n_{2D}q\mu }+\frac{L_{GS}+L_{GD}}{n_{2D0}q\mu_{0}}$$

**Figure 5:**
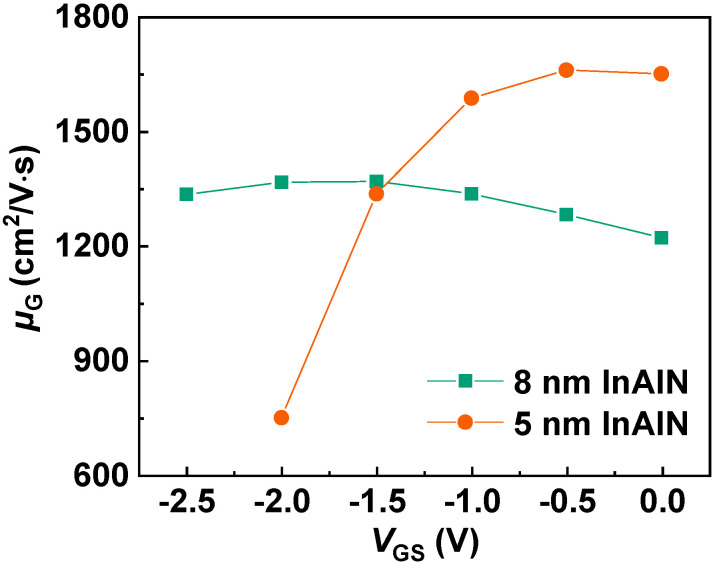
Extracted *µ* versus *V*_GS_ of the devices with 8 nm and 5 nm InAlN.

Figure 5 depicts the extracted *µ* versus *V*_GS_ for both samples. At *V*_GS_ = 0 V, *µ* of the devices with 8 nm InAlN and 5 nm InAlN is 1221 and 1651 cm^2^/V∙s, respectively. The improved electron mobility with a thinner barrier is also confirmed with the Hall measurement (1242 cm^2^/V∙s for 8 nm InAlN and 1663 cm^2^/V∙s for 5 nm InAlN) and the electron mobility of Fat-FETs (with *L*_G_ of 96 µm and *L*_SD_ of 100 µm, 1101 cm^2^/V∙s for 8 nm InAlN and 1670 cm^2^/V∙s for 5 nm InAlN) [29].

**Figure 6:**
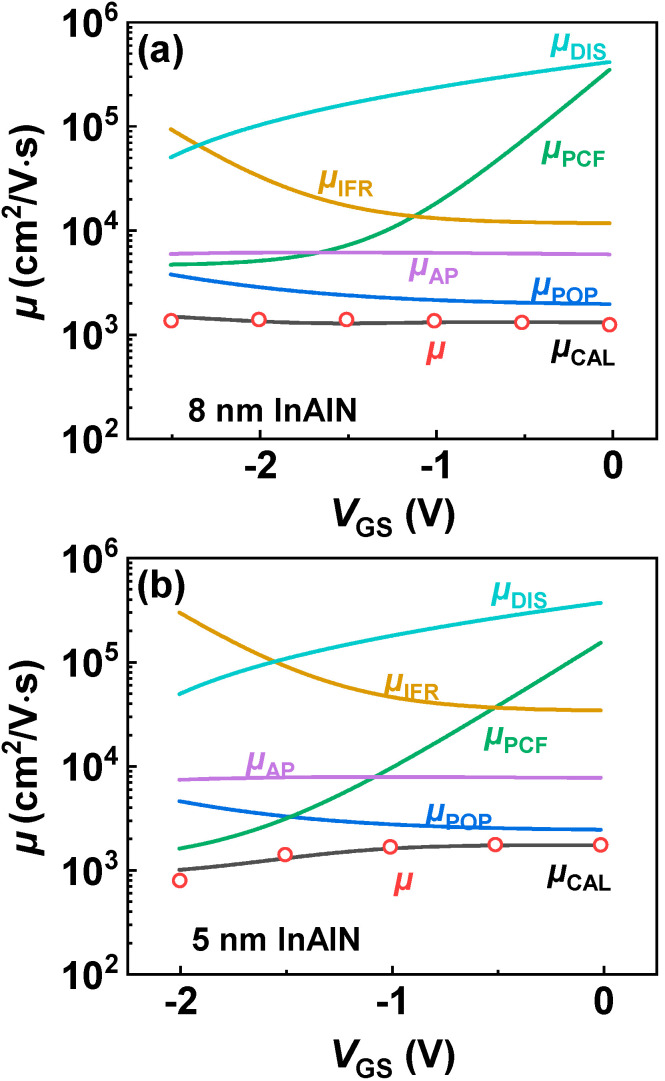
(**a**,**b**) Calculated *µ* limited by different scattering mechanisms, extracted *µ* (*µ*, scatters), and calculated *µ* (*µ*_CAL_, lines) versus *V*_GS_ of both samples.

**Figure 7:**
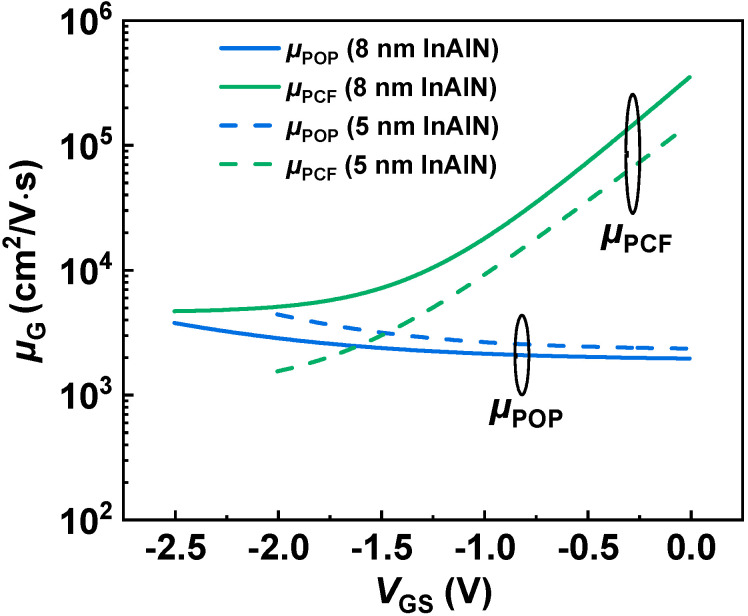
Comparison of *µ*_POP_ and *µ*_PCF_ versus *V*_GS_ of both samples.

**Figure 8:**
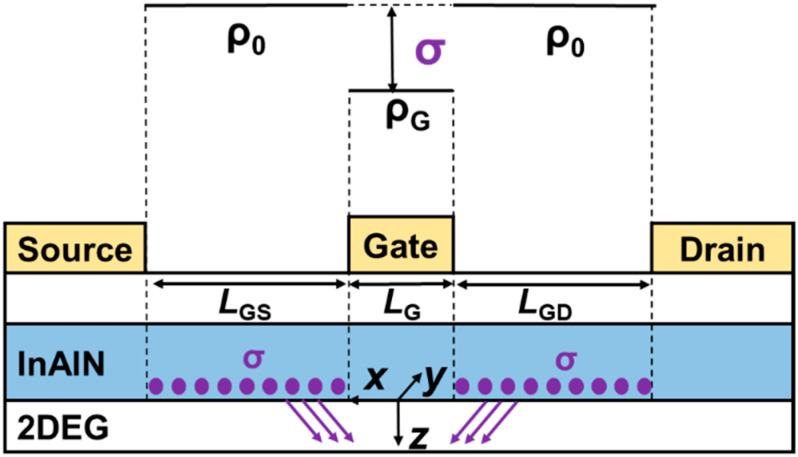
Schematic of the additional polarization charge (σ) distribution in InAlN barrier.

**Figure 9:**
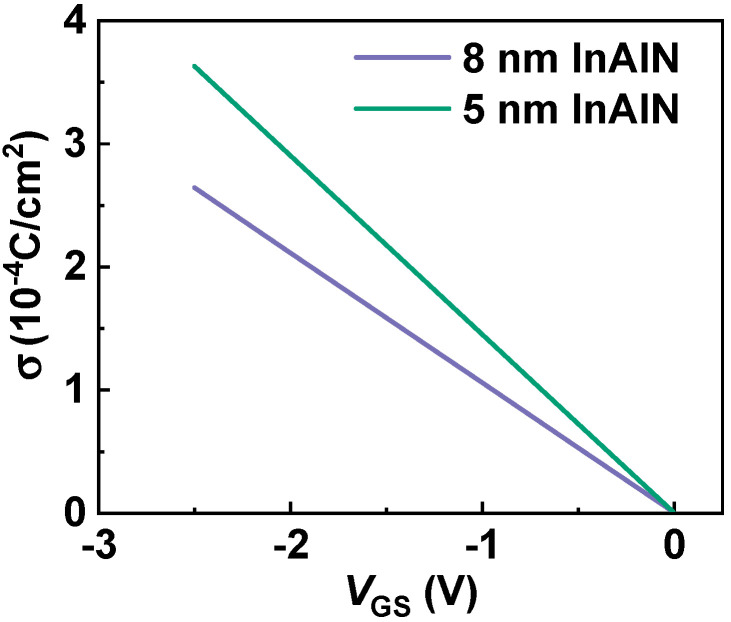
Additional polarization charge (σ) versus *V*_GS_ with 8 nm and 5 nm InAlN barrier.

As shown in Figure 5, *µ* presents a different trend versus *V*_GS_ for the devices with different InAlN thickness. As *V*_GS_ increases, *µ* of the device with 8 nm InAlN deceases, but that of the device with 5 nm InAlN increases. Figure 6a,b show the calculated *µ* limited by different scatterings for both devices [21,30,31]. The calculated *µ* (*µ*_CAL_, lines in the figures) by using 2D scattering theory shows good agreement with the extracted *µ* (scatters in the figures), which proves the accuracy of the calculation. As *V*_GS_ increases, *µ*_POP_ and *µ*_IFR_ decrease, *µ*_DIS_ and *µ*_PCF_ increase, and *µ*_AP_ presents a slight change. *µ*_POP_ and *µ*_PCF_ play more significant roles among all the scatterings. Figure 7 compares *µ*_POP_ and *µ*_PCF_ for both devices. When the InAlN barrier decreases from 8 nm to 5 nm, *µ*_POP_ increases while *µ*_PCF_ decreases. The reduced *n*_2D_ with a 5 nm InAlN barrier decreases the collision probability between channel electrons and polar optical phonons (POPs), resulting in the improved *µ*_POP_ [27,28]. Due to the spontaneous polarization, there are polarization charges (ρ_0_) in the InAlN barrier near the InAlN/GaN interface. When *V*_GS_ is applied on the gate terminal, the polarization charges (ρ_G_) under the gate region are changed due to the inverse piezoelectric effect [32], as shown in Figure 8. The polarization charge distribution is not uniform, and the potential periodicity is broken, resulting in polarization Coulomb field (PCF) scattering. The PCF scattering potential is from the additional polarization charges (σ = ρ_0_ − ρ_G_) and is written as [21,22]

(3) $$\begin{array}{ll} V(x,y,z)= & -\frac{q}{4\pi \varepsilon }\int_{-L_{GS}-\frac{L_{G}}{2}}^{\frac{L_{G}}{2}}dx^{\prime }\int_{0}^{W_{G}}\frac{\sigma }{\sqrt{{\left(x-x^{\prime }\right)}^{2}+{\left(y-y^{\prime }\right)}^{2}+z^{2}}}dy^{\prime }\\ & \;\;-\frac{q}{4\pi \varepsilon }\int_{\frac{L_{G}}{2}}^{\frac{L_{GD}}{2}+\frac{L_{G}}{2}}dx^{\prime }\int_{0}^{W_{G}}\frac{\sigma }{\sqrt{{\left(x-x^{\prime }\right)}^{2}+{\left(y-y^{\prime }\right)}^{2}+z^{2}}}dy^{\prime } \end{array}$$

where *ε* is the dielectric constant of GaN and *W*_G_ is the gate width. Based on inverse piezoelectric effect, σ can be calculated by using σ = ρ_0_ − ρ_G_ = −*ne*_33_^2^*V*_GS_/(*C*_33_*d*) [32]. *n* is the fitting parameter, and *e*_33_ and *C*_33_ are the piezoelectric coefficient and the elastic stiffness tensor of InAlN, respectively. *d* is the gate-to-channel distance, which is the sum of the thicknesses of the GaN cap layer (2 nm), InAlN barrier (8 or 5 nm), and AlN interlayer (1 nm). Figure 9 depicts the calculated σ versus *V*_GS_ with an 8 and 5 nm InAlN barrier. σ increases with the decreased *T*_B_ and *V*_GS_, resulting in the enhanced PCF scattering as the InAlN barrier thickness and *V*_GS_ decrease. Therefore, *µ*_PCF_ increases with *V*_GS_. Because the device with a 5 nm InAlN barrier shows an enhanced PCF scattering, *µ* increases with *V*_GS_. This fact is more pronounced, especially in the more negative *V*_GS_ region. For the device with an 8 nm InAlN barrier, the PCF scattering became weaker and the POP scattering dominates *µ*, leading to a slight decrease in *µ* when *V*_GS_ increases.

**Figure 10:**
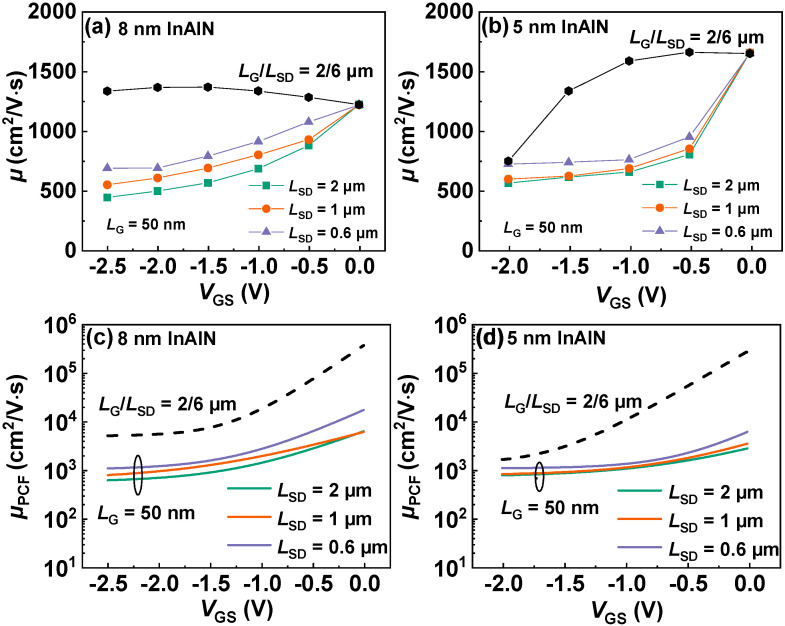
*µ* versus *V*_GS_ for the devices with *L*_G_ of 50 nm and *L*_SD_ of 2, 1, 0.6 µm with (**a**) 8 nm and (**b**) 5 nm InAlN. The device with *L*_G_/*L*_SD_ of 2/6 µm is also plotted for comparison. Calculated *µ*_PCF_ versus *V*_GS_ of the same devices with (**c**) 8 nm and (**d**) 5 nm InAlN. The device with *L*_G_/*L*_SD_ of 2/6 µm is also plotted for comparison.

**Figure 11:**
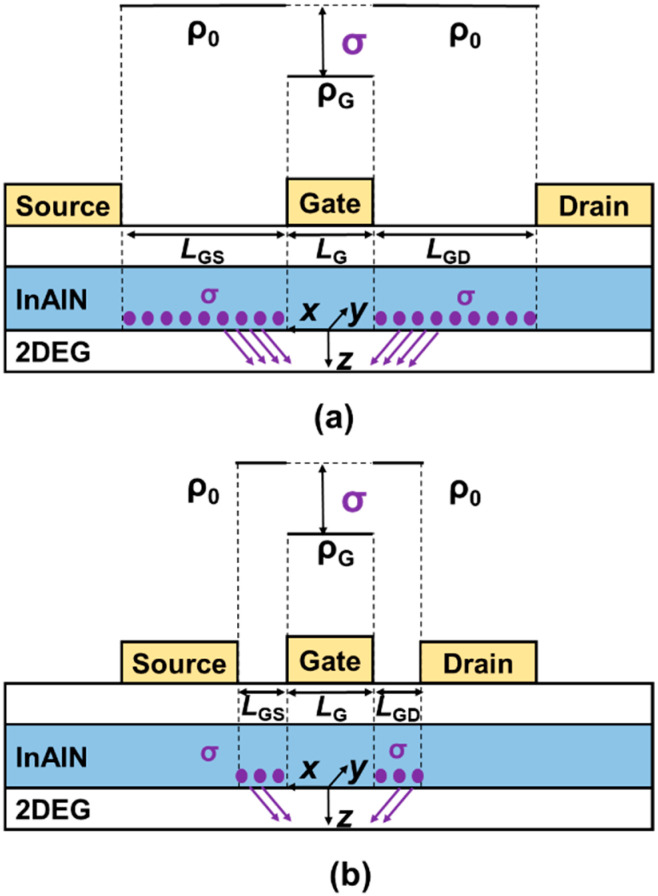
Schematic of the additional polarization charge (σ) distribution in InAlN barrier with (**a**) large and (**b**) small source–drain spacing *L*_SD_. The gate length is fixed.

From the above discussions, the vertical scale will increase σ and thus enhance PCF scattering, leading to a reduced *µ*. The lateral scaling is also experimentally investigated on the devices by varying *L*_SD_ and *L*_G_ using the same electron mobility extraction methodology. As the device laterally scales, *n*_2D_ is not changed, so POP, AP, IFR, and DIS scatterings are not affected. Only PCF scattering can be changed due to the modulation of the polarization change distribution. Figure 10a,b present *µ* versus *V*_GS_ at *V*_DS_ = 0.1 V for the devices with *L*_G_ of 50 nm and *L*_SD_ of 2, 1, 0.6 µm with 8 nm and 5 nm InAlN. *µ* presents an increase with the decrease in *L*_SD_. The corresponding *µ*_PCF_ is also calculated and plotted in Figure 10c,d. As shown in Figure 11a,b, as *L*_SD_ scales down, the number of σ is reduced and the effect of σ on the electron under the gate region is weakened, resulting in the increased *µ*_PCF_ and *µ*. Because PCF scattering in the device with 8 nm InAlN is weaker, the increase in *µ* due to the downscaling of *L*_SD_ is more significant. Here, *µ* of the devices with *L*_G_ of 2 µm is also plotted for comparison, and a significant decrease in *µ* in the device with an *L*_G_ of 50 nm is observed. Although the number of σ is the same under the same *V*_GS_, the effect of σ on the 50 nm gate is stronger and thus PCF scattering is enhanced, leading to a decreased *µ*.

**Figure 12:**
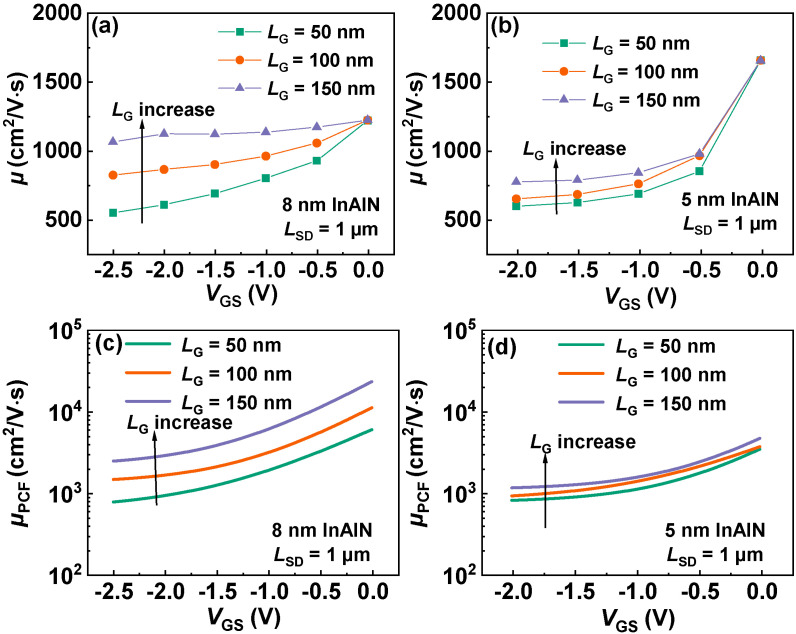
(**a**,**b**) *µ* versus *V*_GS_ for the devices with *L*_SD_ of 1 µm and *L*_G_ of 50, 100, 150 nm with 8 nm and 5 nm InAlN. (**c**,**d**) Calculated *µ*_PCF_ versus *V*_GS_ of the same devices with 8 nm and 5 nm InAlN.

**Figure 13:**
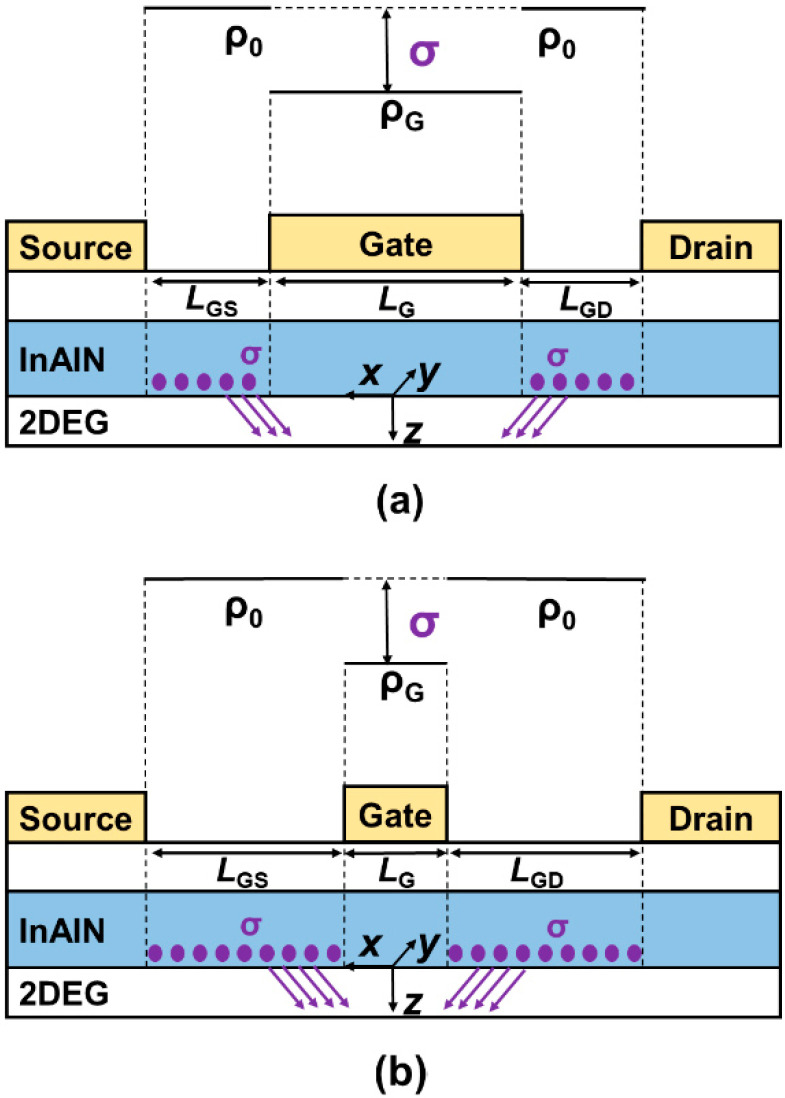
Schematic of the additional polarization charge (σ) distribution in InAlN barrier with (**a**) large and (**b**) small gate length *L*_G_. The source–drain spacing *L*_SD_ is fixed.

Figure 12a,b present the *µ* versus *V*_GS_ for the devices with *L*_SD_ of 1 µm and *L*_G_ of 50, 100, 150 nm with 8 nm and 5 nm InAlN. The electron mobility of all devices presents an increase with *V*_GS_. This means PCF scattering plays a dominant role in the electron mobility. As *V*_GS_ increases from a negative value to 0 V, the electric field under the gate region decreases, resulting in the increase in *µ*_PCF_ and *µ*. For the devices with different gate lengths, *µ* presents an increase as *L*_G_ increases. This means the increase in gate length can increase the electron mobility. To explain this phenomenon, the corresponding *µ*_PCF_ is calculated and plotted in Figure 12c,d. It shows that the increase in *L*_G_ can weaken PCF scattering and increase *µ*_PCF_. Because *L*_SD_ is fixed, as shown in Figure 13, the decreased *L*_G_ means the increased *L*_GS_ and *L*_GD_, resulting in the enhanced effect of σ on the electrons under the gate region. Thus, PCF scattering becomes stronger and *µ* reduces with the downscaled *L*_G_.

## 4. Conclusions

In summary, the effect of down-scaling on electron mobility is experimentally demonstrated. It shows that the downscaling of barrier thickness and *L*_G_ results in a decrease in *µ*, but downscaled *L*_SD_ leads to an increase in *µ*. This is because the polarization charge distribution is modulated with the vertical and lateral scale, resulting in a change in PCF scattering. When GaN HEMTs scale down, the effect of PCF scattering should be considered, providing an insightful guidance for the device geometry design and performance improvement for RF application.

## Data Availability

The data presented in this study are available upon request from the corresponding author.
